# c-FLIP and the NOXA/Mcl-1 axis participate in the synergistic effect of pemetrexed plus cisplatin in human choroidal melanoma cells

**DOI:** 10.1371/journal.pone.0184135

**Published:** 2017-09-01

**Authors:** Xiaofei Zhao, Feng Kong, Lei Wang, Han Zhang

**Affiliations:** 1 Department of Ophthalmology, the Second Hospital of Shandong University, Jinan, Shandong Province, China; 2 Department of Central Research Laboratory, the Second Hospital of Shandong University, Jinan, Shandong Province, China; Columbia University, UNITED STATES

## Abstract

Choroidal melanoma is the most common primary malignant intraocular tumor, and very few effective therapies are available to treat it. Our study aimed to understand whether pemetrexed plus cisplatin exerts a beneficial synergistic effect in human choroidal melanoma cells and to delineate the underlying molecular mechanism. To accomplish these aims, we treated choroidal melanoma cells with pemetrexed and cisplatin and assessed cell survival with SRB and MTT assays. Proteins were detected using western blotting analysis. NOXA and CHOP were knocked down with siRNA. We found that pemetrexed or cisplatin alone inhibited survival and induced apoptosis in human choroidal melanoma cells. Furthermore, the expression levels of c-FLIP, an anti-apoptotic protein in the extrinsic apoptosis pathway, and Mcl-1, an anti-apoptotic protein in the intrinsic apoptosis pathway, were decreased by pemetrexed or cisplatin respectively, while the expression of a pro-apoptotic protein in the intrinsic apoptosis pathway, NOXA, was up-regulated. Moreover, pemetrexed or cisplatin alone increased the protein expression of the endoplasmic reticulum stress markers IRE1α, Bip and CHOP. Silencing CHOP expression reduced NOXA expression. These findings suggest that the pemetrexed or cisplatin induced intrinsic apoptosis via activation of the ER stress response. Importantly, combining the two compounds more strongly induced apoptosis. Following the cotreatment, CHOP and NOXA expression increased, while c-FLIP and Mcl-1 expression decreased, and these effects were more pronounced than when using either compound alone. This result suggests that pemetrexed and cisplatin synergistically activate ER stress response-induced apoptosis in choroidal melanoma cells. To summarize, the c-FLIP and NOXA/Mcl-1 axis participated in the synergistic effect of pemetrexed plus cisplatin in human choroidal melanoma cells. Intrinsic apoptosis was induced via activation of the ER stress response. Our study provides important mechanistic insights into potential cancer treatment with pemetrexed plus cisplatin and enriches our understanding of human choroidal melanoma.

## Introduction

Choroidal melanoma is the most common primary malignant tumor of the eye, accounting for approximately 70% of all primary intraocular eye tumors [1], and the second most common type of malignant melanoma affecting the body. Approximately 50% of choroidal melanomas metastasize within 15 years of the primary diagnosis [2]. Once metastasis has occurred, the median survival time is less than 12 months [3]. Due to this high risk for metastasis, the prognosis of choroidal melanoma is poor [4].

Choroidal melanoma is insensitive to many commonly used chemotherapeutic drugs [5], and there is currently no routinely used conventional chemotherapy for these tumors [6]. Present treatments for choroidal melanoma, such as enucleation, focal radiotherapy and transpupillary thermotherapy, offer only temporary relief and are ineffective in inhibiting tumor metastasis or improving the survival rate [2, 5, 7]. Therefore, there is significant interest in developing better therapies for this condition.

Cisplatin is a frequently used chemotherapeutic drug that plays an anti-cancer role by binding to genomic DNA, causing DNA damage and ultimately leading to cell death [8–10]. Previous studies have suggested that cisplatin induces cell death by down-regulating the telomerase activity of choroidal melanoma cells in a dose- and time-dependent manner [11].

In many clinical cases, cisplatin is combined with other drugs to reduce adverse side effects and achieve better therapeutic activity [8]. Pemetrexed, a recently developed anti-folate compound with favorable toxic effects, is well tolerated by patients and has shown cytotoxicity against breast and lung cancer cell lines [12]. Cisplatin has been combined with pemetrexed to treat advanced lung adenocarcinoma and malignant mesothelioma with strong efficacy and reduced adverse events [13, 14]. However, the molecular mechanism underlying the synergistic effect of these two compounds is still unclear. Determining whether the combination of pemetrexed and cisplatin exhibit a positive synergistic effect on choroidal melanoma and understanding the underlying molecular mechanism is important for developing better chemotherapeutics for this condition.

In the present study, we explored whether the combination of pemetrexed plus cisplatin exerts a beneficial synergistic effect on choroidal melanoma cells. We also explored the role of c-FLIP and NOXA as regulators of this synergistic effect. Our study reveals the therapeutic potential of pemetrexed plus cisplatin for choroidal melanoma and enriches our understanding of the disease.

## Materials and methods

### Antibodies and reagents

Cisplatin was purchased from Sigma-Aldrich (St. Louis, MO, USA) and dissolved in normal saline (NS) at a concentration of 5 mmol/L [15]. Pemetrexed was purchased from Toronto Research Chemicals (Toronto, ON, Canada) and dissolved in dimethyl sulfoxide (DMSO) at a concentration of 10 mmol/L [16]. Aliquots were stored at -20°C, and stock solutions were diluted to the desired concentrations with growth medium before use. All antibodies used have been previously described [17]. The antibodies casp8, casp9, PARP, IRE1α and Bip were purchased from Cell Signaling Technology (Danvers, MA, USA). Casp3 antibody was obtained from Imgenex (San Diego, CA, USA). Actin antibody was purchased from Sigma-Aldrich (St. Louis, MO, USA). Antibodies against CHOP and c-FLIP were obtained from Santa Cruz (Santa Cruz, CA, USA) and Alexis (San Diego, CA, USA), respectively. NOXA antibody was purchased from Calbiochem (Merck KGaA, Darmstadt, Germany).

### Cell lines and cell culture

Human choroidal melanoma cells were obtained from the China Center for Type Culture Collection (Wuhan, China) and were grown in monolayer culture at 37°C in a humidified atmosphere consisting of 5% CO_2_ and 95% air. M619 cells were cultured in RPMI 1640 medium containing 5% fetal bovine serum, and OCM1 cells were cultured in Dulbecco's modified Eagle's medium.

### Cell survival assay

Cells were seeded in 96-well microtiter plates and then treated with the indicated concentrations of cisplatin or pemetrexed on the second day. After this, the cells were cultured for another 36 hours and then subjected to SRB (sulforhodamine B) or MTT assay. To assay the synergistic effect of the combination of pemetrexed and cisplatin, the cells were treated with both drugs at various concentrations for 24 hours. The live cell number was estimated as previously described [17]. For the SRB assays, the medium was first discarded after treatment, and then, 100 μl of cold trichloroacetic acid (10% (w/v)) was added to each well followed by incubation at 4°C for at least 1 hour to fix any adherent cells. The plates were then washed five times with deionized water and air-dried. Following this, 50 μl of SRB solution (0.4% w/v in 1% acetic acid) was added to each well, and the plate was incubated at room temperature for 5 min. To remove unbound SRB, the plate was washed five times with 1% acetic acid. After air-drying the wells, 100 μ1 of 10 mM Tris base buffer (pH 10.5) was added to solubilize the residual bound SRB in each well, and the plates were read using a microtiter plate reader at 495 nm. For the MTT assay, 20 μl of MTT (5 mg/ml) was added to each sample followed by incubation at 37°C for 4 hours. Following this, the solution was discarded, and 100 μl of dimethyl sulfoxide was added. Cell viability was determined at 495 nm.

### Western blotting analysis

Whole-cell protein lysates were prepared and analyzed with western blotting according to a previously described protocol [17]. Cells were harvested and rinsed with pre-chilled PBS then, cells were lysed and the extract was centrifuged at 4°C for 15 minutes. The resultant whole-cell protein lysates (40 μg) were electrophoresed through 12% denaturing polyacrylamide slab gels and then transferred to a Hybond enhanced chemiluminescence (ECL) membrane by electroblotting. Proteins were probed with the appropriate primary antibodies and subsequently with secondary antibodies. Antibody binding was detected using an ECL system (Millipore, Billerica, MA, USA) according to the manufacturer’s protocol.

### Isobologram analysis

The synergistic effect of pemetrexed and cisplatin was analyzed using an isobologram analysis according to a previously described method [18]. Cells were treated with the combination of pemetrexed and cisplatin at various concentrations. The IC50 values of the individual components and the combination were calculated using GraphPad Prism 5.0 software. X-axes represented for cisplatin concentration while y-axes represented for pemetrexed concentration. An isobole was constructed by joining the points that represented the IC50 values of pemetrexed and cisplatin. If the dots that represent the IC50 of the combination fall on this isobole, it represents an additive effect, whereas dots falling underneath the isobole line represent a synergistic effect, and dots falling above the isobole represent an antagonistic effect.

### Apoptosis assay

Apoptosis was evaluated according to a previously described protocol [17] using an Annexin V-FITC/PI apoptosis detection kit purchased from BIO-BOX Biotech (Nanjing, China). Briefly, 2×10^6^ cells were harvested and washed with pre-chilled PBS and then resuspended in 500 μl of binding buffer. Then, 5 μl of Annexin V-FITC and 5 μl of propidium iodide (PI) were added to each sample, and the tube was incubated for 10 minutes in the dark at room temperature. Analysis was performed using a FACScan flow cytometer (Becton Dickinson, San Jose, CA, USA).

### Transfection with siRNA

Previously described siRNAs targeting sequences of CHOP and NOXA were synthesized [17]. SiRNA transfection was conducted using jetPRIME Transfection Reagent (Polyplus Transfection SA, Illkirch, France) following the manufacturer’s protocol. Cells were seeded in 6-well plates and transfected with control or target siRNA on the second day. Two days after the transfection, the cells were treated with the indicated concentration of pemetrexed or cisplatin for another 36 hours or the combination of pemetrexed and cisplatin for 24 hours. Following this, the cells were harvested for western blotting and apoptosis analysis.

### Statistical analysis

The data obtained represent the mean and standard deviation (SD)of at least three independent assays performed in duplicate or triplicate. An unpaired t-test was used for significance testing for all assays. P < 0.05 was considered statistically significant.

## Results

### Pemetrexed and cisplatin inhibited the survival of human choroidal melanoma cells by inducing apoptosis

**Fig 1:**
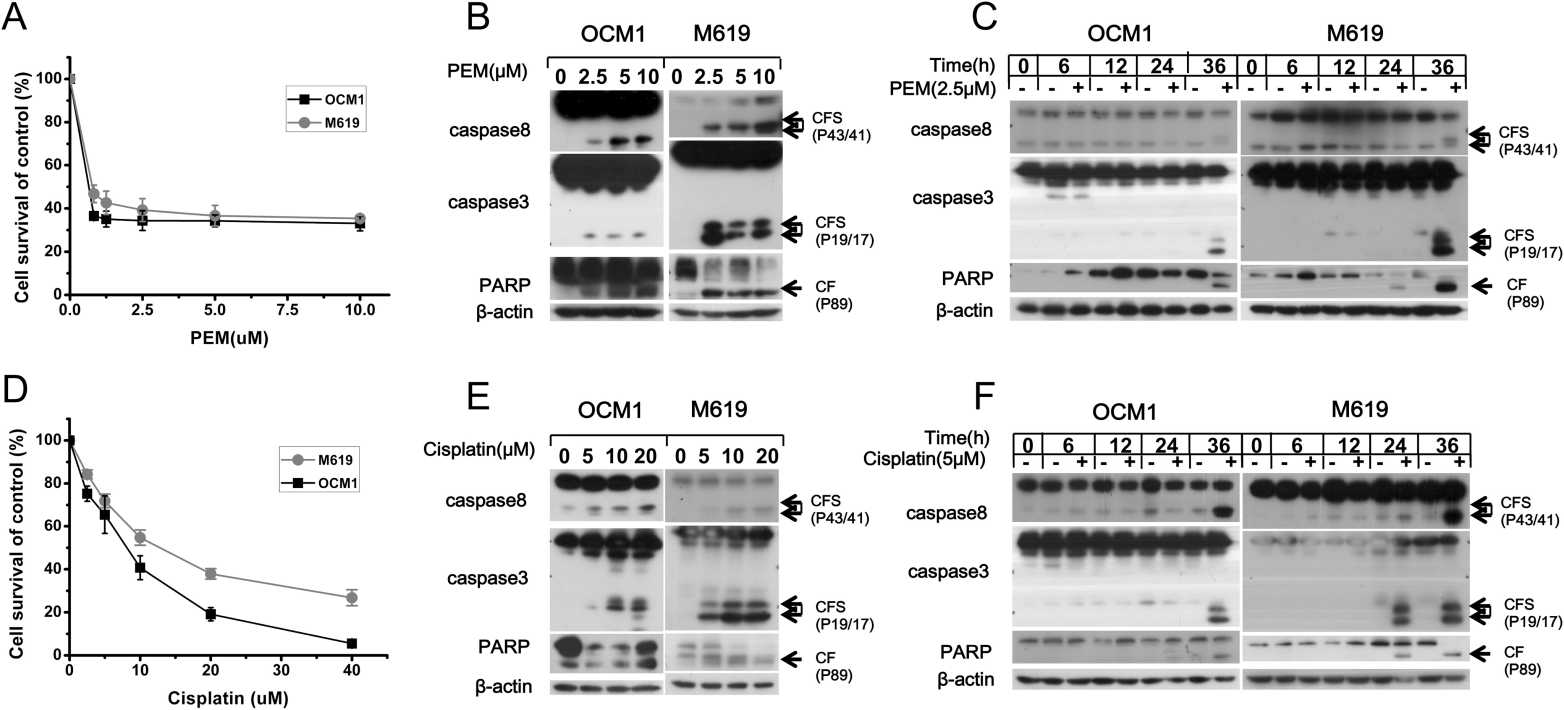
Pemetrexed and cisplatin inhibit cell growth and induce apoptosis in a concentration-dependent and time-dependent manner. (A, D) OCM1 and M619 cells were seeded in 96-well plates and treated with increasing doses of pemetrexed or cisplatin for 36 hours. Cell survival was estimated using an SRB assay. The expression of proteins related to apoptosis in the pemetrexed or cisplatin group was examined by western blotting. (B, E) To perform a dose-gradient assay, cells were treated with increasing doses of pemetrexed or cisplatin for 36 hours. (C, F) To perform a time-gradient assay, cells were treated with 2.5 μmol/L pemetrexed or 5 μmol/L cisplatin for the indicated time, then, western blotting analysis was used to determine the expression levels of apoptosis-related proteins. CF: cleaved form. CFs: plural of cleaved form. All data are presented as the mean ± S.D.

To examine the cytotoxicity of pemetrexed and cisplatin in choroidal melanoma cells, OCM1 and M619 cells were treated with various concentrations of the drugs for 36 hours and then submitted to SRB assay. The results showed that pemetrexed weakly inhibited cell survival in a dose-dependent manner (Fig 1A). To describe the molecular mechanism underlying this effect, we used western blotting analysis to detect the ability of the chemotherapeutic agent to induce apoptosis. The data showed that pemetrexed induced cleavage and activation of the apoptotic proteins casp8, casp3 and PARP in a concentration- and time-dependent manner (Fig 1B and 1C). Similar results were obtained when cells were treated with cisplatin (Fig 1D, 1E and 1F). Overall, these findings indicate that both pemetrexed and cisplatin can trigger apoptosis.

### Low-dose pemetrexed plus cisplatin has a synergistic effect on human choroidal melanoma cells

**Fig 2:**
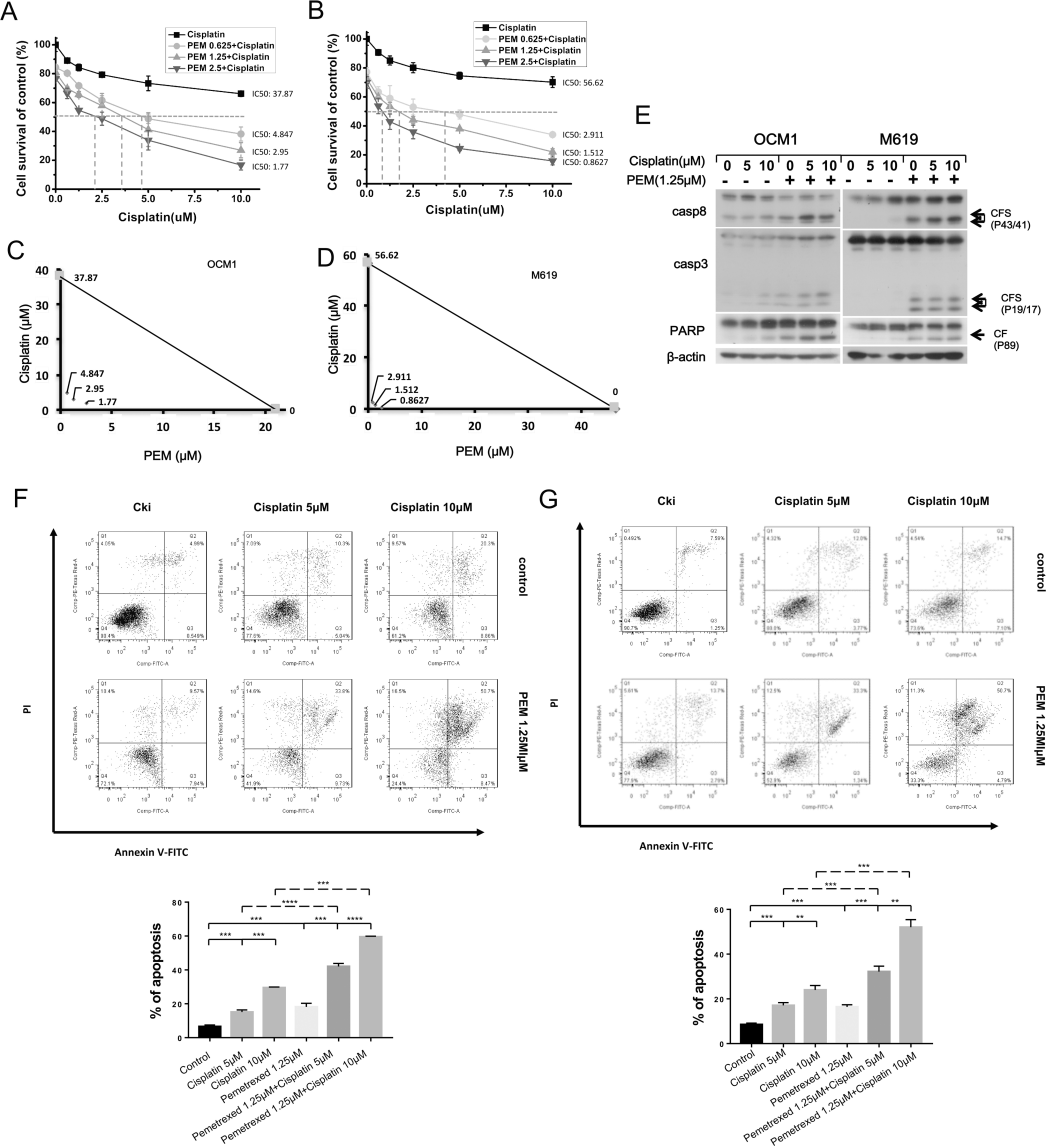
Low-dose pemetrexed plus cisplatin synergistically work to inhibit survival and induce apoptosis in human choroidal melanoma cells. (A) OCM1 and (B) M619 cells were seeded in 96-well plates and treated with increasing doses of pemetrexed combined with various doses of cisplatin respectively for 24 hours. The IC50 values for the individual components and the drug combination were calculated using GraphPad Prism 5.0 software. The IC50 values for pemetrexed and cisplatin were 21.04μM and 37.87μM respectively while the values were 4.847μM, 2.95μM and 1.77μM for various concentrations of drug combination respectively in OCM1 cells. Meanwhile, the IC50 values for pemetrexed and cisplatin were 46.38μM and 56.62μM respectively and the values were 2.911μM, 1.512μM and 0.8627μM for various concentrations of drug combination respectively in M619 cells. (C, D) The synergistic effect of pemetrexed and cisplatin was analyzed using isobolograms. X-axes represented for cisplatin concentration while y-axes represented for pemetrexed concentration. An isobole was constructed by joining the points that represented the IC50 values of pemetrexed and cisplatin, and the dots underlying the isobole represented the IC50 values of the combination treatment. (E, F, G) OCM1 and M619 cells were treated with various concentrations of the chemotherapeutic drugs for 24 hours and then harvested for western blotting and apoptosis analysis. CF: cleaved form. CFs: plural of cleaved form. All data are presented as the mean ± S.D.

The combination of pemetrexed plus cisplatin has been reported to have a synergistic effect in various tumor types; therefore, we examined the cytotoxicity of this combination in choroidal melanoma cells. Cells treated with pemetrexed or cisplatin alone for 24 hours did not exhibit satisfactory therapeutic effects (S1 Fig), leading us to examine whether the combination of the two drugs could achieve better therapeutic activity. To accomplish this, we treated OCM1 and M619 cells with various concentrations of cisplatin alone or in combination with different doses of pemetrexed for 24 hours, after which MTT and isobologram assays were executed. An isobole was constructed by joining the points that represented the IC50 values of pemetrexed and cisplatin, and the dots represented the IC50 values of the combination treatment were all underlying the isobole which meant that low doses of pemetrexed in combination with cisplatin synergistically inhibited the survival of human choroidal melanoma cells (Fig 2A, 2B, 2C and 2D). Western blotting showed that this combination induced greater cleavage of the apoptotic proteins casp8, casp3 and PARP (Fig 2E). Furthermore, Annexin V staining analysis showed that the combination more strongly induced apoptosis than either drug alone (Fig 2F and 2G). Overall, these results show that a low dose of pemetrexed in combination with cisplatin strongly induces apoptosis in choroidal melanoma cells.

### c-FLIP is down-regulated in choroidal melanoma cells following the induction of extrinsic apoptosis by pemetrexed plus cisplatin

Because c-FLIP is an important modulator of the extrinsic apoptotic pathway, we measured c-FLIP expression levels after the drug treatment and found that both the long form (c-FLIP_L_) and short form (c-FLIPs) of c-FLIP were down-regulated in a concentration- and time-dependent manner (S2 Fig). These results indicate that c-FLIP is involved in the induction of extrinsic apoptosis by cisplatin and pemetrexed when these compounds are used individually.

**Fig 3:**
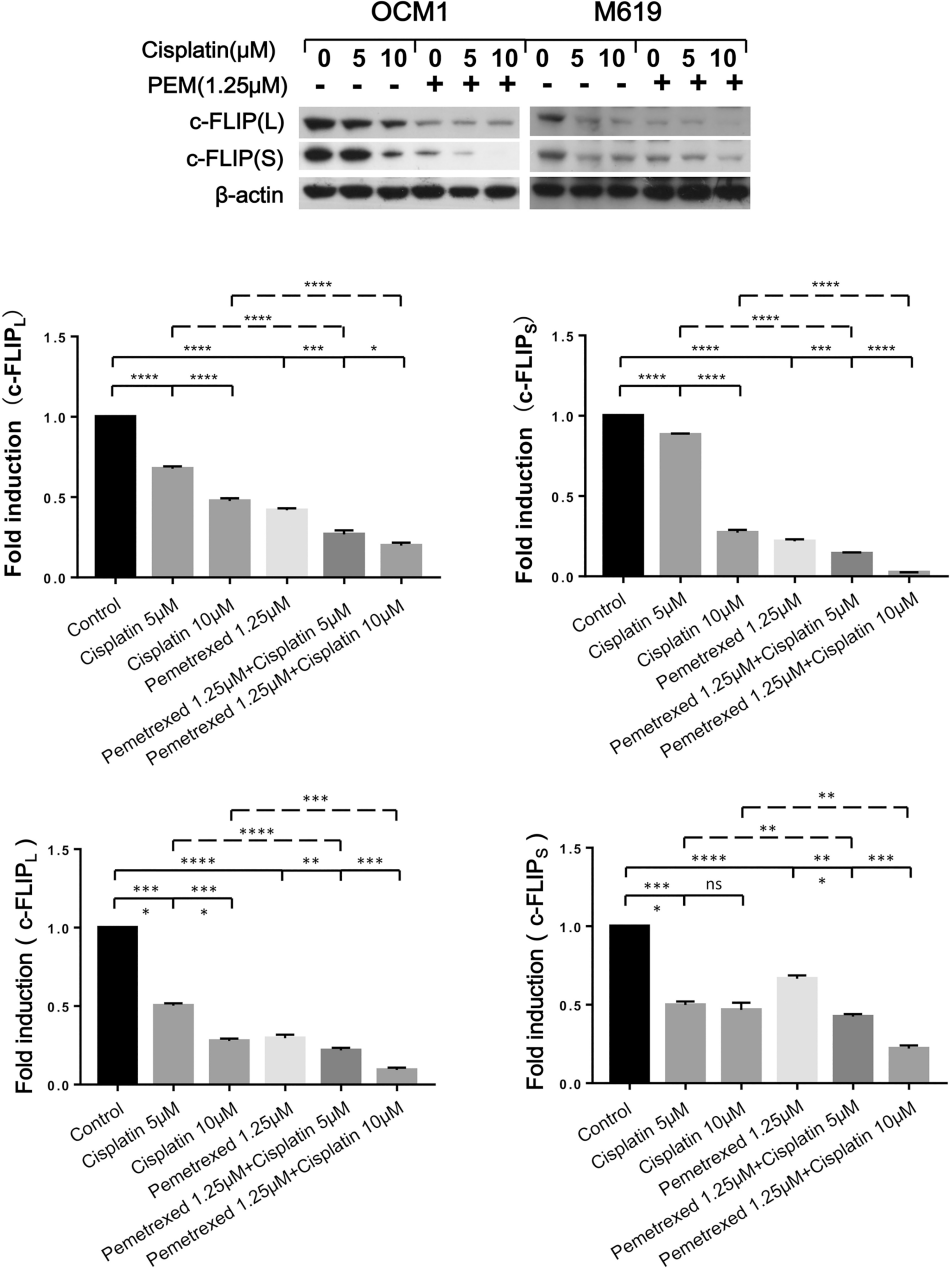
Low-dose pemetrexed plus cisplatin has a synergistic effect in reducing c-FLIP expression in choroidal melanoma cells. OCM1 and M619 cells were treated with 1.25μM pemetrexed combined with increasing doses of cisplatin respectively for 24 hours and then harvested for western blotting analysis. The expression of c-FLIP was quantified using Image J software and analyzed with GraphPad Prism 5.0 software. All data are presented as the mean ± S.D.

We further examined whether c-FLIP has a role in the synergistic effect of pemetrexed plus cisplatin. The results showed that the combination more strongly reduced c-FLIP_L_ and c-FLIPs (Fig 3) levels than either of the drugs alone, indicating that c-FLIP does have a role in the synergy attained between the two drugs in choroidal melanoma cells.

### NOXA and Mcl-1 contribute to the induction of intrinsic apoptosis following treatment with pemetrexed plus cisplatin in choroidal melanoma cells

To discover whether the combination of pemetrexed and cisplatin induces the intrinsic apoptotic pathway in choroidal melanoma cells, we detected the expression of proteins important in the intrinsic apoptotic signaling pathway after drug treatment. Western blotting analysis revealed that NOXA was up-regulated in both a concentration- and time-dependent manner after drug exposure, while Mcl-1 was down-regulated (S3A, S3B, S3C and S3D Fig). When expression of NOXA was knocked down using siRNA, the protein level of Mcl-1 was partially increased, and the cleavage of pro-caspases and PARP that was induced by pemetrexed and cisplatin was reduced compared with the control (S3E and S3F Fig). Analysis of Annexin V staining showed that apoptosis was inhibited when NOXA was knocked down (S3G–S3J Fig). Overall, these results show that pemetrexed and cisplatin regulates the NOXA and Mcl-1 signaling axis to induce intrinsic apoptosis.

**Fig 4:**
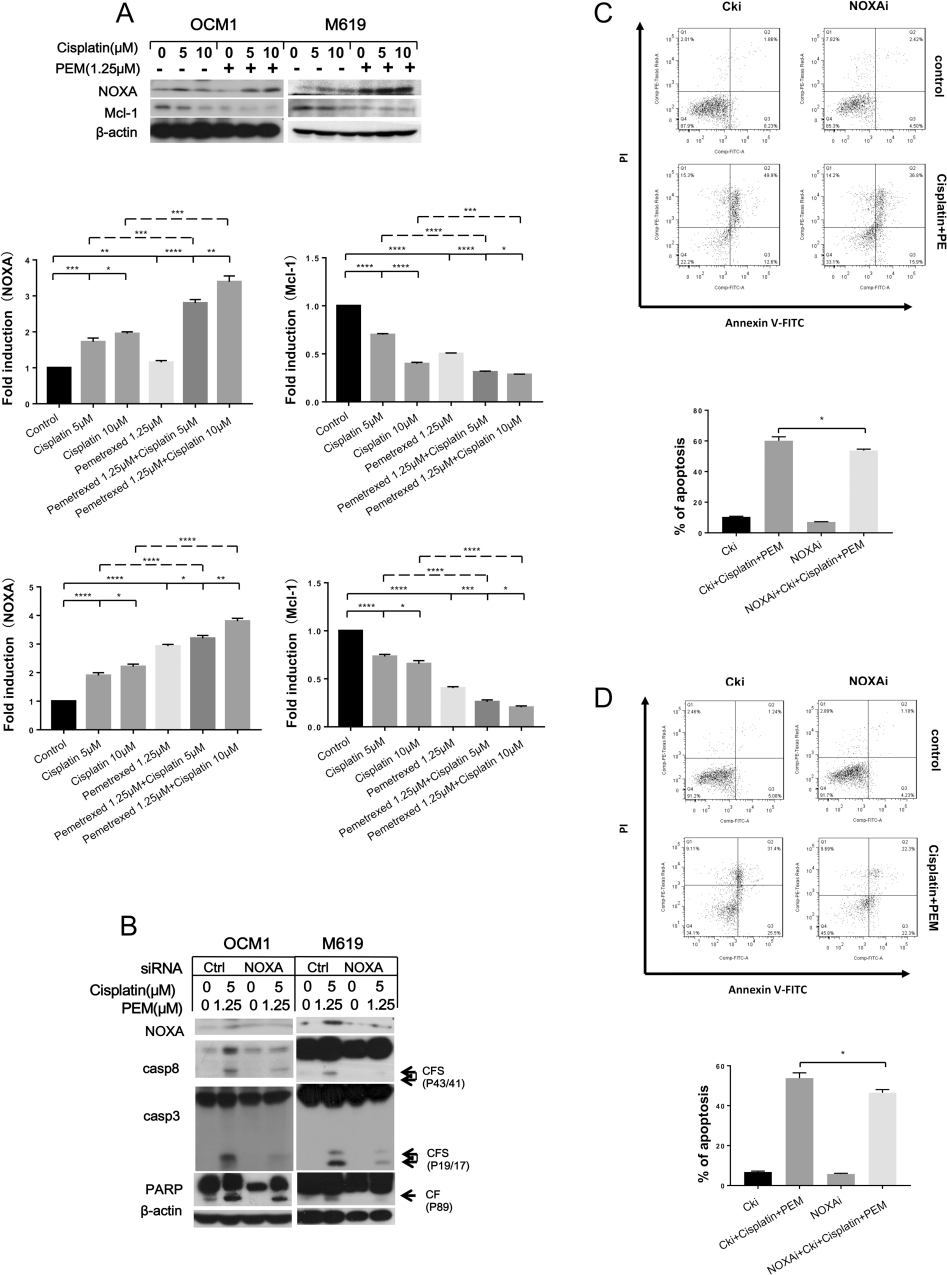
Low-dose pemetrexed plus cisplatin synergistically up-regulates NOXA expression and down-regulates Mcl-1 expression in choroidal melanoma cells. (A) To analyze the synergistic effect of the co-treatment, the indicated cells were treated with the indicated concentrations of the chemotherapeutic drugs for 24 hours and then harvested for western blotting. NOXA and Mcl-1 expression was quantified using Image J software and analyzed with GraphPad Prism 5.0 software. OCM1 and M619 cells were seeded in 6-well plates and transfected with control or NOXA siRNA on the second day. (B, C, D)At 48 hours after transfection, the cells were treated with 1.25 μmol/L pemetrexed combined with 5 μmol/L cisplatin for another 24 hours and then harvested for western blotting and apoptosis analysis. CF: cleaved form. All data are presented as the mean ± S.D.

Furthermore, we examined whether NOXA and Mcl-1 had a role in the synergistic effect produced by the combined treatment. The results showed that the combination more strongly reduced expression of the anti-apoptotic protein Mcl-1 than either drug alone and robustly up-regulated NOXA (Fig 4A). When the expression of NOXA was knocked down and cells were treated with pemetrexed combined with cisplatin, the protein level of Mcl-1 partially increased, and the cleavage of pro-caspases and PARP that was induced by the drugs was reduced compared with the control (Fig 4B). Analysis of Annexin V staining showed that apoptosis was inhibited when NOXA was knocked down (Fig 4C and 4D). Therefore, we conclude that NOXA and Mcl-1 contribute to the synergistic effect of the co-treatment in choroidal melanoma cells.

### CHOP regulates the induction of NOXA expression and apoptosis by pemetrexed and cisplatin

Because NOXA is a target gene of CHOP, we next examined whether CHOP has a role in inducing NOXA expression in choroidal melanoma cells after drug treatment. Western blotting revealed that CHOP expression increased in both a concentration- and time-dependent manner after drug treatment (S4A, S4B, S4C and S4D Fig). When CHOP was knocked down, the expression of NOXA decreased, and the activation of pro-caspases was simultaneously weakened compared with control siRNA-transfected cells (S4E and S4F Fig). Analysis of Annexin V staining showed that less apoptosis was induced by pemetrexed and cisplatin after knock down of CHOP (S4G–S4J Fig).

**Fig 5:**
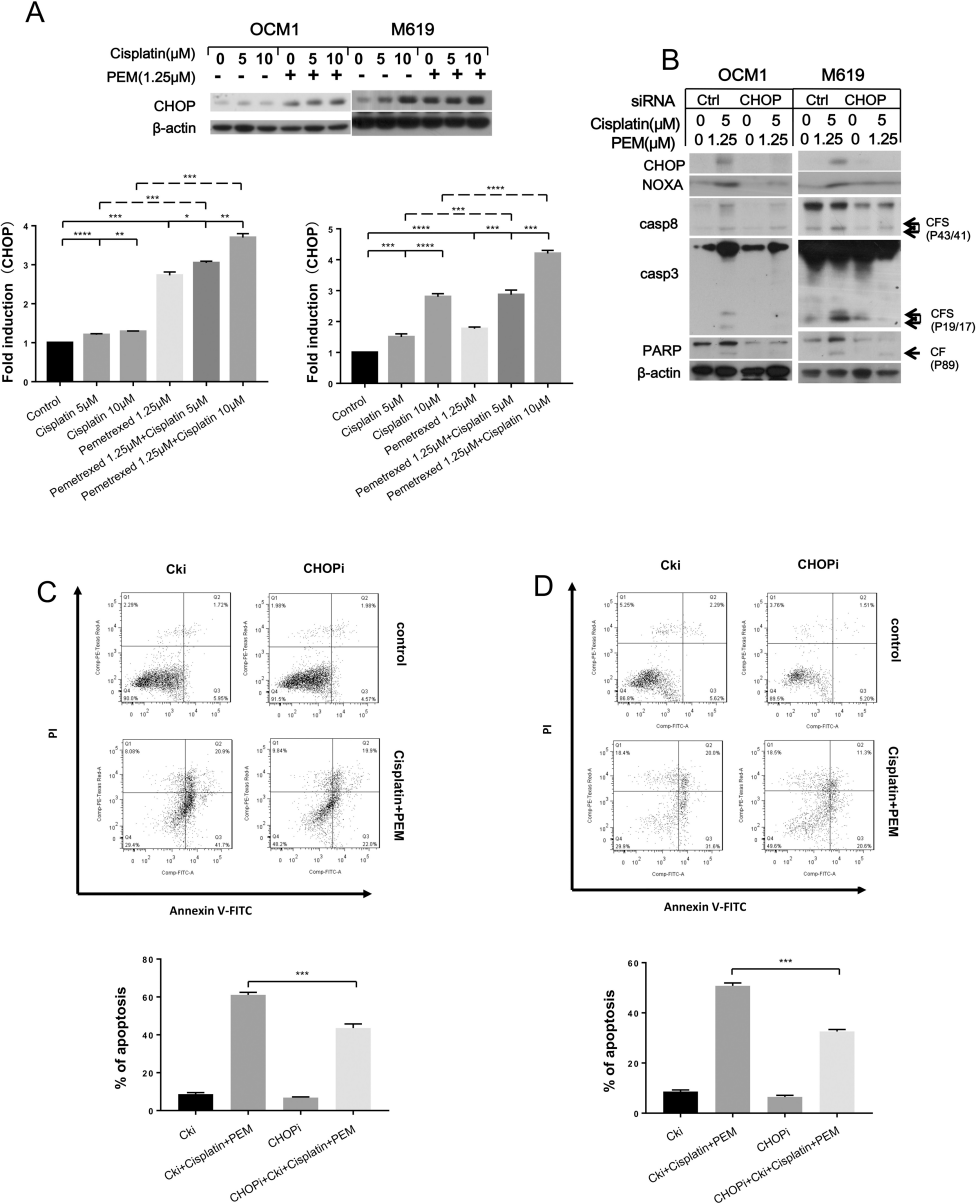
Pemetrexed plus cisplatin synergistically induces CHOP expression. (A) To analyze the synergistic effects of the co-treatment, the indicated cells were treated with the indicated concentrations of chemotherapeutic drugs for 24 hours and then harvested for western blotting analysis. CHOP expression was quantified using Image J software and analyzed with GraphPad Prism 5.0 software. OCM1 and M619 cells were seeded in 6-well plates and transfected with control or CHOP siRNA on the second day. (B, C) At 48 hours after transfection, the cells were treated with 1.25 μmol/L pemetrexed combined with 5 μmol/L cisplatin for another 24 hours and then harvested for western blotting and apoptosis analysis. CF: cleaved form. All data are presented as the mean ± S.D.

We further examined CHOP expression after exposure to pemetrexed plus cisplatin. The results showed that pemetrexed in combination with cisplatin more strongly induced CHOP expression (Fig 5A). When CHOP expression was knocked down and cells were treated with pemetrexed combined with cisplatin, the protein level of NOXA was partially increased, and the cleavage of pro-caspases and PARP that was induced by the drugs was reduced compared with the control (Fig 5B). Analysis of Annexin V staining showed that apoptosis was inhibited when CHOP was knocked down (Fig 5C and 5D). Therefore, we conclude that CHOP contributes to the synergistic effect of pemetrexed plus cisplatin by regulating NOXA expression in choroidal melanoma cells.

**Fig 6:**
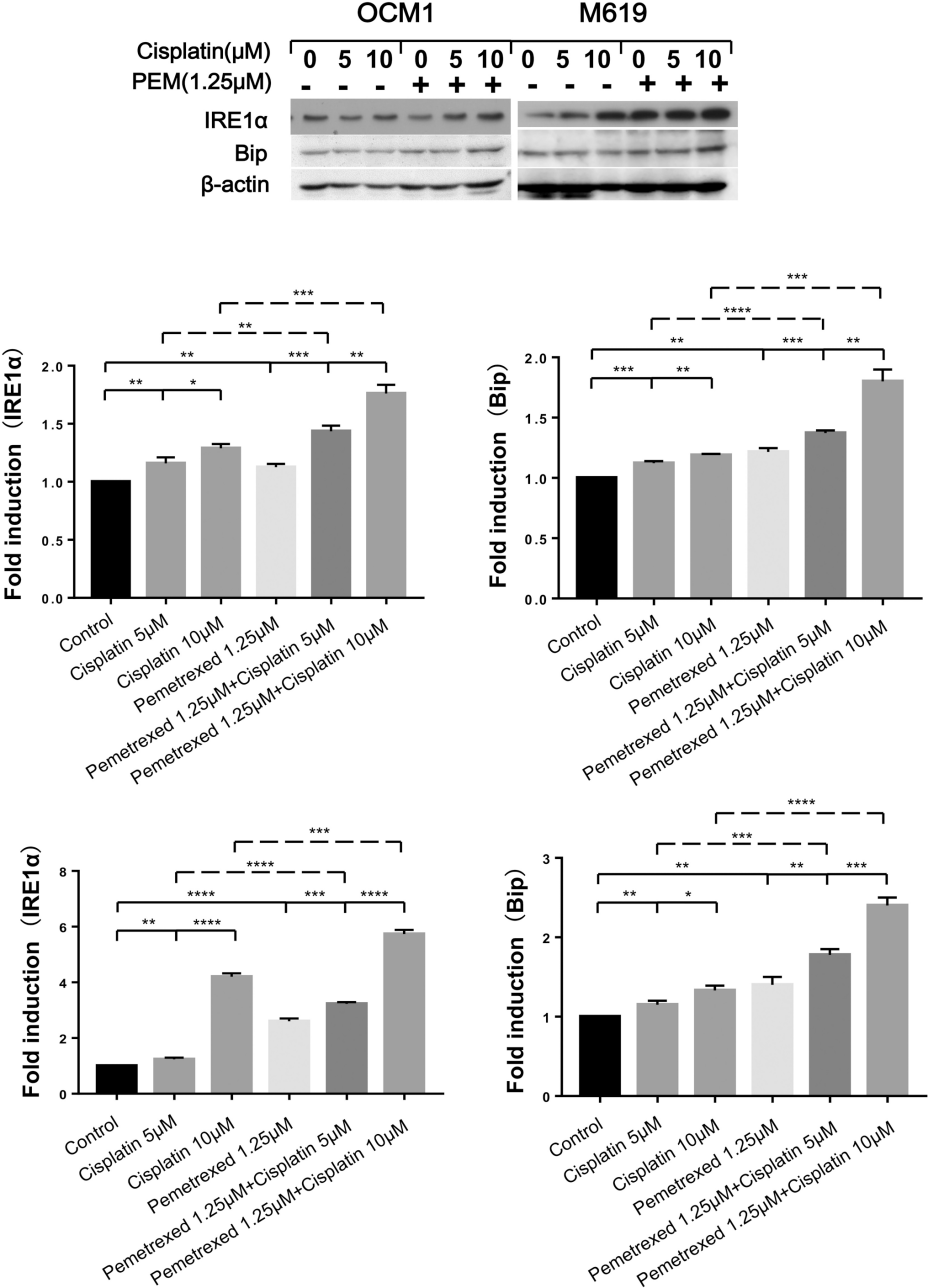
ER stress-related proteins are clearly up-regulated following treatment with pemetrexed in combination with cisplatin. To analyze the synergistic effects of the co-treatment, the indicated cells were treated with the indicated concentrations of chemotherapeutic drugs for 24 hours and then harvested for western blotting analysis. IRE1α and Bip expression was quantified using Image J software and analyzed with GraphPad Prism 5.0 software. All data are presented as the mean ± S.D.

Because CHOP is an important hallmark of the endoplasmic reticulum (ER) stress pathway, we examined the expression of IRE1α and Bip, two molecules involved in ER stress signaling, after drug exposure [19]. The results demonstrated that both molecules were up-regulated by the drugs in a concentration- and time-dependent manner (S5A, S5B, S5C and S5D Fig). We also found that the combination of pemetrexed and cisplatin more strongly induced IRE1α than Bip, indicating that a high degree of ER stress was produced (Fig 6). Overall, these results indicate that pemetrexed and cisplatin induce apoptosis through ER stress response signaling. Low-dose pemetrexed in combination with cisplatin can induce robust ER stress and strongly trigger apoptosis in choroidal melanoma cells.

## Discussion

Choroidal melanoma is the most frequently occurring primary intraocular malignant tumor and is always accompanied by metastasis and poor survival [1]. In addition, the cancer is insensitive to many commonly used chemotherapeutic drugs. All existing therapies offer only temporary relief and do not improve patient survival. Thus, there is currently no effective therapy for this disease [2, 4].

Cisplatin is a commonly used chemotherapeutic drug[8], while pemetrexed is a recently developed compound that has been reported to have a favorable toxic effect against gastric cancer cell lines[12]. The combination of pemetrexed and cisplatin has been used to treat advanced lung adenocarcinoma and malignant mesothelioma [13, 14]. However, how this combination targets cancer cells and induces apoptosis in choroidal melanoma cells is largely unexplained.

In the current study, we found that pemetrexed and cisplatin alone inhibited the survival of human choroidal melanoma cells and induced apoptosis in a concentration- and time-dependent manner in all tested cells at 36 hours. However, when cells were treated with pemetrexed or cisplatin alone for 24 hours, the same level of inhibition was not observed. When OCM1 and M619 cells were treated with various concentrations of cisplatin in combination with different low doses of pemetrexed for 24 hours, the drugs appeared to produce a synergistic effect with regard to survival inhibition. Whether low-dose cisplatin in combination with various concentrations of pemetrexed could produce same synergistic effect and the underlying molecular mechanism require further research.

Subsequently, we found that the drug combination dramatically induced apoptosis. Apoptosis is governed by an extrinsic death receptor signaling pathway and an intrinsic mitochondrial signaling pathway [20]. c-FLIP, an important anti-apoptotic protein in the extrinsic apoptosis pathway, prevents recruitment of pro-casp8 to DISC, inhibits activation of initiator caspases and subsequently inhibits downstream effectors [21]. To understand the downstream molecular mechanism through which pemetrexed and cisplatin induce apoptosis in choroidal melanoma cells, we measured the expression of apoptosis-related proteins and found that c-FLIP levels decreased after exposure to the drugs in a concentration- and time-dependent manner. When cells were treated with pemetrexed plus cisplatin, c-FLIP was more strongly down-regulated. DR4 and DR5 are also important proteins in the extrinsic apoptotic pathway [22, 23], however, whether they are involved in cisplatin and pemetrexed-induced apoptosis is unclear. Based on the results of the current study, we speculate that c-FLIP is the major responder driving extrinsic apoptosis in choroidal melanoma cells following treatment with pemetrexed plus cisplatin.

The mitochondrial pathway is modulated by members of the Bcl-2 family, which contains both anti-apoptotic and pro-apoptotic proteins [24]. NOXA is a BH3-only protein in the Bcl-2 family that has been reported to participate in chemotherapy-induced apoptosis in melanoma [25]. Mcl-1 is an anti-apoptotic protein that can interact with NOXA, which displaces Bax from the Mcl-1/Bax complex and releases Bax to subsequently induce the mitochondrial apoptotic pathway [26]. This combination of NOXA and Mcl-1 can also promote proteasomal degradation of Mcl-1 and enhance mitochondrial apoptosis [27].

We also examined proteins involved in the intrinsic apoptotic pathway. NOXA expression increased after drug treatment, while Mcl-1 expression decreased. Knockdown of NOXA resulted in up-regulation of Mcl-1 and reduced cleavage of caspases. The anti-apoptotic protein Mcl-1 was more strongly down-regulated by the combination than either drug alone, while NOXA expression was further enhanced. Overall, our findings indicate that the NOXA/Mcl-1 axis contributes to the intrinsic apoptosis induced by pemetrexed plus cisplatin in choroidal melanoma cells. Bcl-2 is another member of the intrinsic apoptotic pathway and has been reported to be highly expressed in uveal melanoma. Furthermore, a compound targeting Bcl-2/Bcl-XL may have anti-tumor activity [28]. Combined with our results, this observation suggests that further exploration focusing on potential chemical compounds targeting NOXA may provide better therapeutic options for treating human choroidal melanoma.

Numerous chemotherapeutic agents have been reported to induce ER stress in tumor cells [29]. Persistent ER stress eventually triggers apoptosis [30]. CHOP is an ER stress effector and functions as a bZIP-containing transcription factor that targets several apoptotic genes, including NOXA, regulating their expression and finally resulting in apoptosis [31].

We examined CHOP expression in choroidal melanoma cells after treatment with pemetrexed and cisplatin. Our results showed that CHOP was up-regulated after exposure to the drugs, and CHOP knockdown resulted in down-regulation of NOXA and less apoptosis compared with the control. Subsequently, we examined IRE1α and Bip, two other molecules involved in ER stress, and found that both were up-regulated by pemetrexed and cisplatin in a concentration- and time-dependent manner. Altogether, these results indicate that pemetrexed and cisplatin induce apoptosis in choroidal melanoma cells through activation of the ER stress response. The ER stress proteins IRE1α, Bip, and CHOP were also found to be more strongly up-regulated by the combination treatment than treatment with either drug alone, indicating that the combination more strongly induced the ER stress response. This stronger ER stress response further increased NOXA expression, eventually leading to apoptosis.

## Conclusions

In summary, we demonstrated that pemetrexed and cisplatin induced both extrinsic and intrinsic apoptosis in human choroidal melanoma cells. Intrinsic apoptosis was induced via activation of the ER stress response. Moreover, the combination of pemetrexed and cisplatin induced intense ER stress and apoptosis in these cells. Our study provides important mechanistic insight into a potential cancer treatment using these two compounds and enriches our understanding of human choroidal melanoma.

## Supporting information

S1 Fig Pemetrexed and cisplatin inhibit cell growth in a time-dependent manner. (A, B) OCM1 and (C, D) M619 cells were treated with increasing doses of pemetrexed or cisplatin at various time points and then harvested for MTT assays. All data are presented as the mean ± S.D.
(TIF)

S2 Fig c-FLIP is down-regulated in cells undergoing pemetrexed- and cisplatin-induced apoptosis in a dose-dependent and time-dependent manner. (A, B) OCM1 and M619 cells were treated with the indicated concentrations of pemetrexed or cisplatin for 36 hours. (C, D) For the time-gradient assay, cells were treated with 2.5 μmol/L pemetrexed or 5 μmol/L cisplatin at various time points and harvested for western blotting analysis.
(TIF)

S3 Fig Pemetrexed and cisplatin induce intrinsic apoptosis by up-regulating NOXA expression and down-regulating Mcl-1 expression in a dose-dependent and time-dependent manner. (A, B) OCM1 and M619 cells were treated with the indicated concentrations of pemetrexed or cisplatin for 36 hours. (C, D) For the time-gradient assay, cells were treated with 2.5 μmol/L pemetrexed or 5 μmol/L cisplatin for various lengths of time and then harvested for western blotting. (E-J) OCM1 and M619 cells were seeded in 6-well plates and transfected with control or NOXA siRNA on the second day. Forty-eight hours after the transfection, the cells were treated with 2.5 μmol/L pemetrexed or 5 μmol/L cisplatin for another 36 hours and then harvested for western blotting and apoptosis analysis. CF: cleaved form. All data are presented as the mean ± S.D.
(TIF)

S4 Fig Pemetrexed and cisplatin induce apoptosis by up-regulating CHOP in a dose-dependent and time-dependent manner. (A, B) OCM1 and M619 cells were treated with the indicated concentrations of pemetrexed or cisplatin for 36 hours. (C, D) For the time-gradient assay, cells were treated with 2.5 μmol/L pemetrexed or 5 μmol/L cisplatin for various lengths of time and then harvested for western blotting analysis. (E-J) OCM1 and M619 cells were seeded in 6-well plates and transfected with control or CHOP siRNA on the second day. At 48 hours after transfection, the cells were treated with 2.5 μmol/L pemetrexed or 5 μmol/L cisplatin for another 36 hours and then harvested for western blotting and apoptosis analysis. CF: cleaved form. All data are presented as the mean ± S.D.
(TIF)

S5 Fig Pemetrexed and cisplatin up-regulate the endoplasmic reticulum markers IRE1α and Bip in a dose-dependent and time-dependent manner. (A, B) OCM1 and M619 cells were treated with the indicated concentrations of pemetrexed or cisplatin for 36 hours. (C, D) For the time-gradient assay, cells were treated with 2.5 μmol/L pemetrexed or 5 μmol/L cisplatin for various lengths of time and then harvested for western blotting analysis.
(TIF)
