# Supplementary figures and images for: c-FLIP and the NOXA/Mcl-1 axis participate in the synergistic effect of pemetrexed plus cisplatin in human choroidal melanoma cells
Source: PLoS One. 2017 Sep 1;12(9):e0184135. doi: 10.1371/journal.pone.0184135 (PMC5581197; doi:10.1371/journal.pone.0184135)

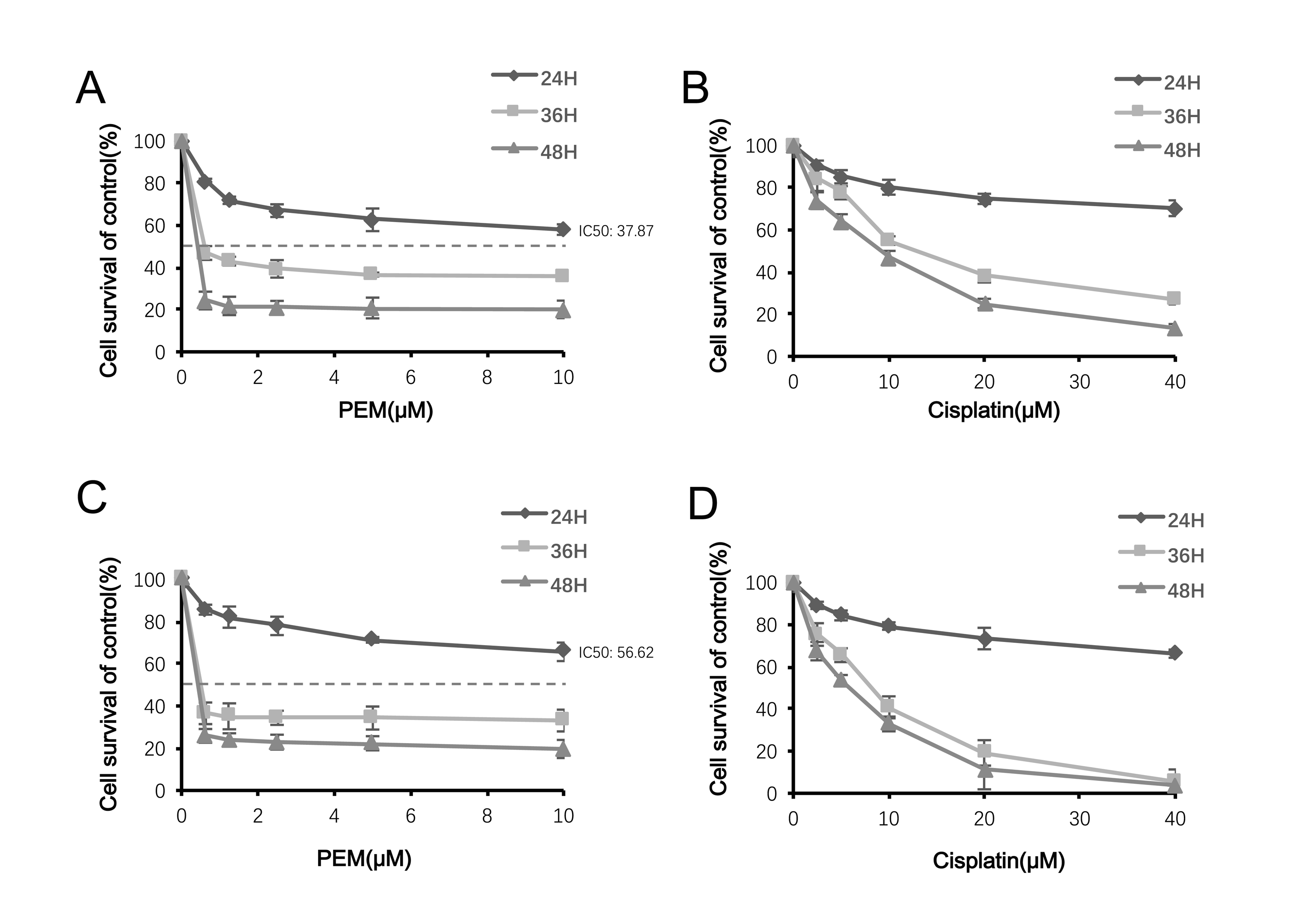

Supplement: S1 Fig — (A, B) OCM1 and (C, D) M619 cells were treated with increasing doses of pemetrexed or cisplatin at various time points and then harvested for MTT assays. All data are presented as the mean ± S.D. (TIF) [file pone.0184135.s001.tif]

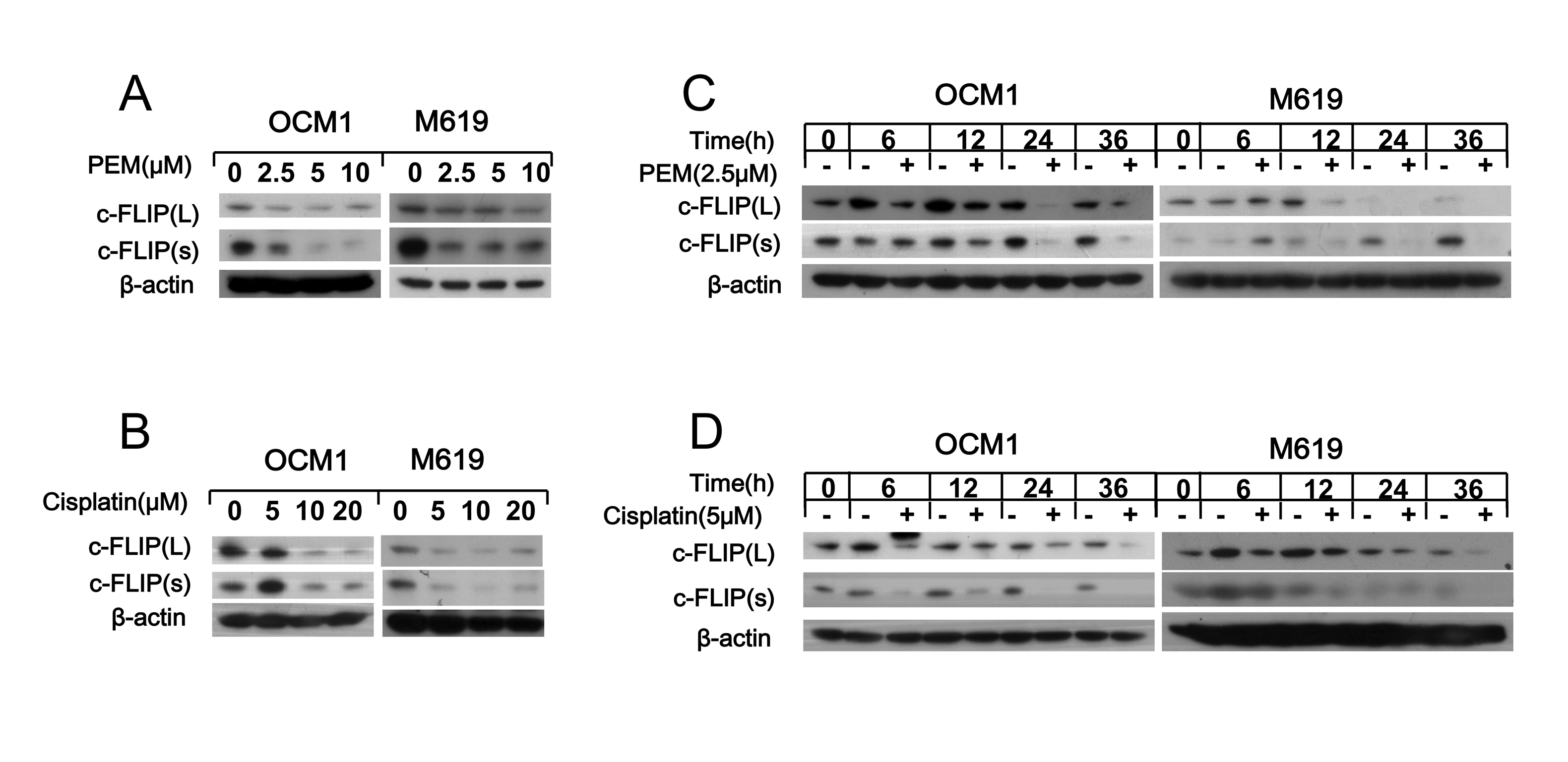

Supplement: S2 Fig — (A, B) OCM1 and M619 cells were treated with the indicated concentrations of pemetrexed or cisplatin for 36 hours. (C, D) For the time-gradient assay, cells were treated with 2.5 μmol/L pemetrexed or 5 μmol/L cisplatin at various time points and harvested for western blotting analysis. (TIF) [file pone.0184135.s002.tif]

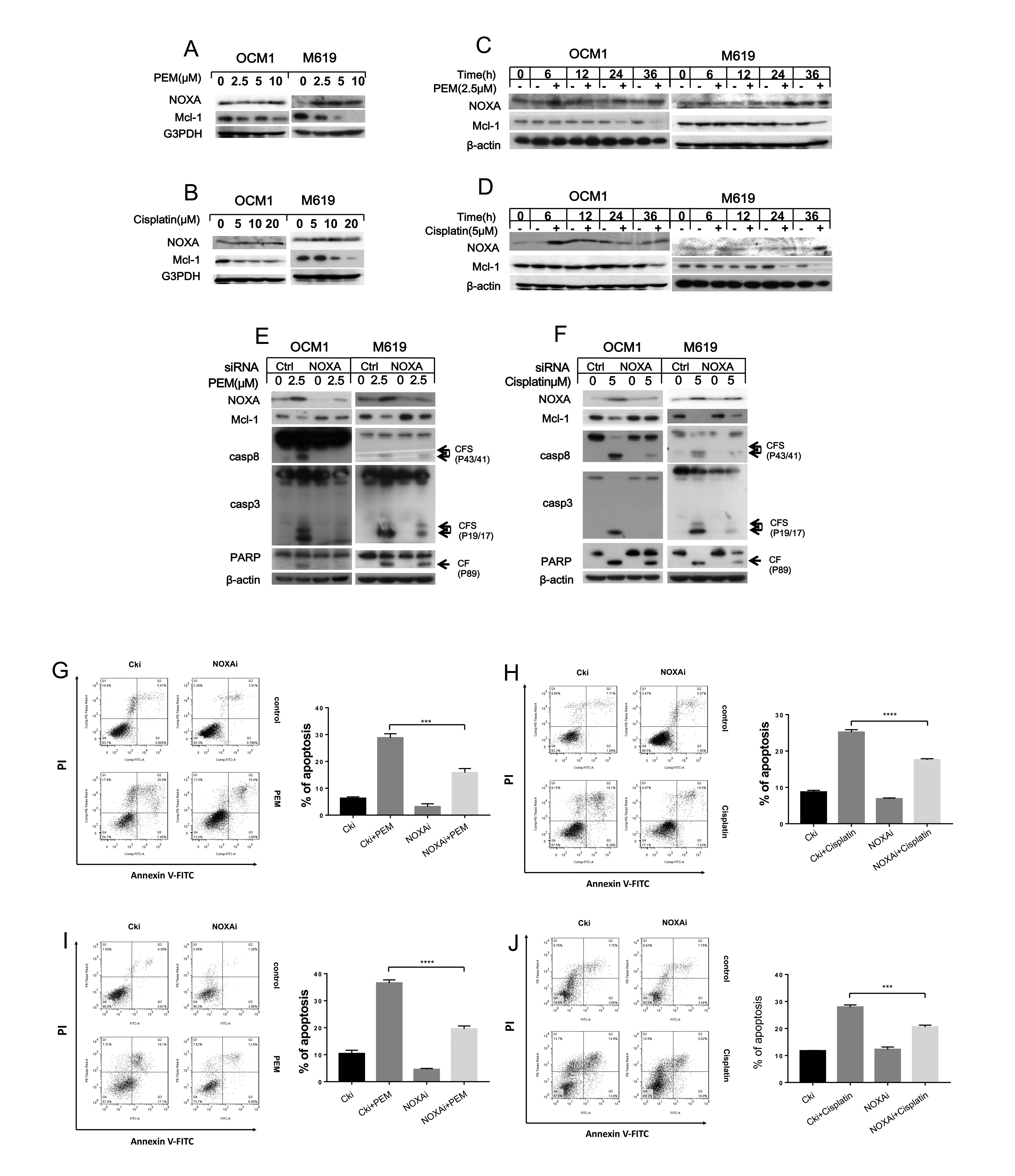

Supplement: S3 Fig — (A, B) OCM1 and M619 cells were treated with the indicated concentrations of pemetrexed or cisplatin for 36 hours. (C, D) For the time-gradient assay, cells were treated with 2.5 μmol/L pemetrexed or 5 μmol/L cisplatin for various lengths of time and then harvested for western blotting. (E-J) OCM1 and M619 cells were seeded in 6-well plates and transfected with control or NOXA siRNA on the second day. Forty-eight hours after the transfection, the cells were treated with 2.5 μmol/L pemetrexed or 5 μmol/L cisplatin for another 36 hours and then harvested for western blotting and apoptosis analysis. CF: cleaved form. All data are presented as the mean ± S.D. (TIF) [file pone.0184135.s003.tif]

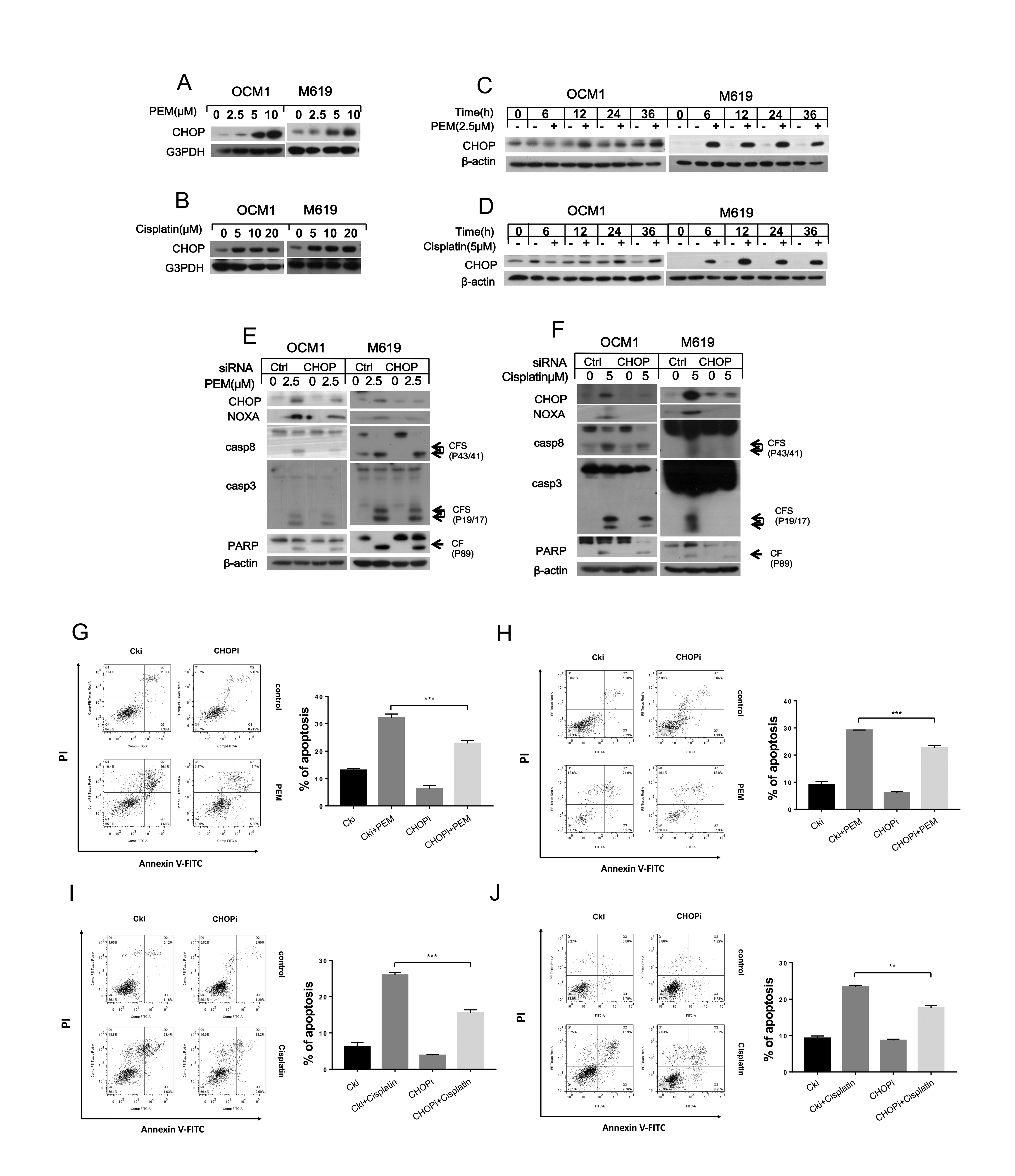

Supplement: S4 Fig — (A, B) OCM1 and M619 cells were treated with the indicated concentrations of pemetrexed or cisplatin for 36 hours. (C, D) For the time-gradient assay, cells were treated with 2.5 μmol/L pemetrexed or 5 μmol/L cisplatin for various lengths of time and then harvested for western blotting analysis. (E-J) OCM1 and M619 cells were seeded in 6-well plates and transfected with control or CHOP siRNA on the second day. At 48 hours after transfection, the cells were treated with 2.5 μmol/L pemetrexed or 5 μmol/L cisplatin for another 36 hours and then harvested for western blotting and apoptosis analysis. CF: cleaved form. All data are presented as the mean ± S.D. (TIF) [file pone.0184135.s004.tif]

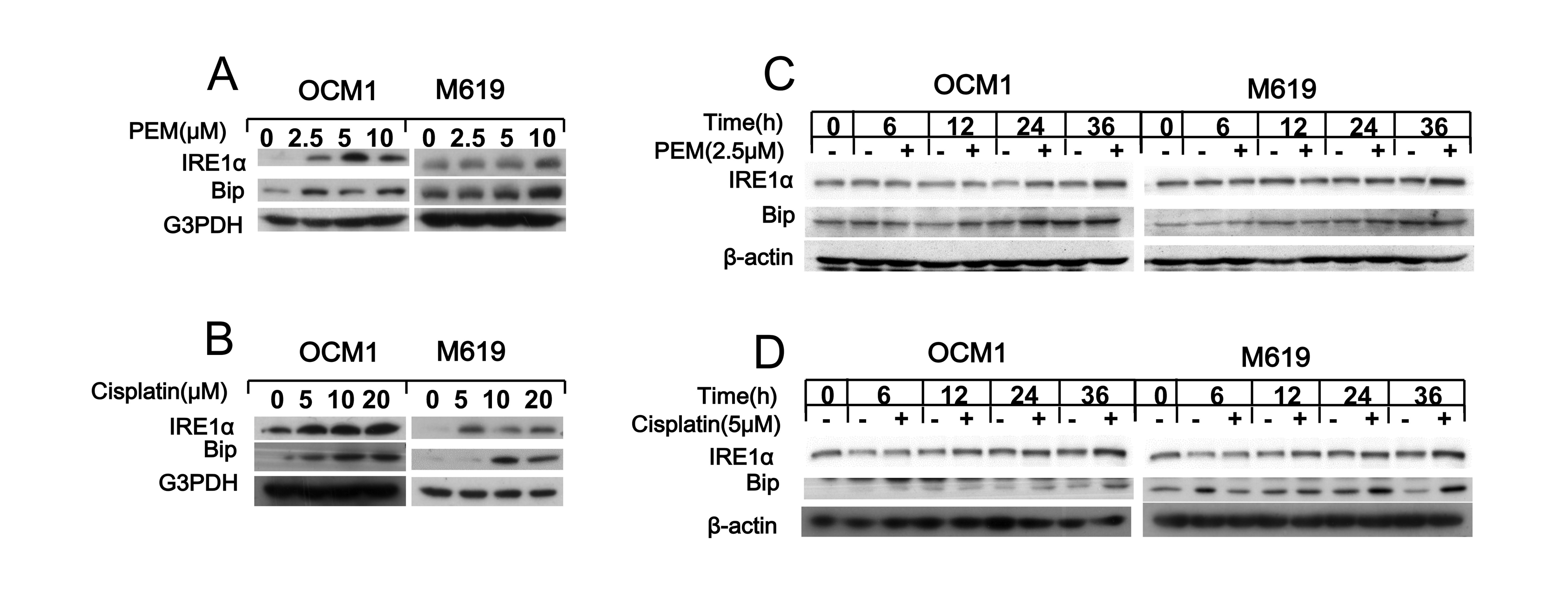

Supplement: S5 Fig — (A, B) OCM1 and M619 cells were treated with the indicated concentrations of pemetrexed or cisplatin for 36 hours. (C, D) For the time-gradient assay, cells were treated with 2.5 μmol/L pemetrexed or 5 μmol/L cisplatin for various lengths of time and then harvested for western blotting analysis. (TIF) [file pone.0184135.s005.tif]
